# Utilization of Eco-Friendly Waste Generated Nanomaterials in Water-Based Drilling Fluids; State of the Art Review

**DOI:** 10.3390/ma14154171

**Published:** 2021-07-27

**Authors:** Rabia Ikram, Badrul Mohamed Jan, Akhmal Sidek, George Kenanakis

**Affiliations:** 1 Department of Chemical Engineering, University of Malaya, Kuala Lumpur 50603, Malaysia; 2 Petroleum Engineering Department, School of Chemical and Energy Engineering, Faculty of Engineering, Universiti Teknologi Malaysia, Johor Bahru 81310, Malaysia; akhmalsidek@utm.my; 3 Institute of Electronic Structure and Laser, Foundation for Research and Technology-Hellas, N. Plastira 100, Vasilika Vouton, GR-700 13 Heraklion, Greece

**Keywords:** waste derivatives, rheological properties, nanomaterials, graphene, drilling fluids

## Abstract

An important aspect of hydrocarbon drilling is the usage of drilling fluids, which remove drill cuttings and stabilize the wellbore to provide better filtration. To stabilize these properties, several additives are used in drilling fluids that provide satisfactory rheological and filtration properties. However, commonly used additives are environmentally hazardous; when drilling fluids are disposed after drilling operations, they are discarded with the drill cuttings and additives into water sources and causes unwanted pollution. Therefore, these additives should be substituted with additives that are environmental friendly and provide superior performance. In this regard, biodegradable additives are required for future research. This review investigates the role of various bio-wastes as potential additives to be used in water-based drilling fluids. Furthermore, utilization of these waste-derived nanomaterials is summarized for rheology and lubricity tests. Finally, sufficient rheological and filtration examinations were carried out on water-based drilling fluids to evaluate the effect of wastes as additives on the performance of drilling fluids.

## 1. Introduction

Drilling operations are conducted to obtain oil and gas from natural reservoirs deep underground [1]. To facilitate the extraction of hydrocarbons from the ground, a deep hole is drilled to form a wellbore. The use of drilling fluids is an important factor in the drilling process, and these fluids play many roles, such as to assist in removal of drill cuttings and formation pressure control [2,3]. There are viable chemical additives used in the drilling fluid that have shown the desired features. However, these additives are non-biodegradable and environmentally hazardous [4]. As a result, researchers have sought to identify alternate additives that are environmentally friendly, biodegradable, and sustainable, while also maintaining the properties of efficient drilling fluids [5].

Drilling fluids must address several challenges encountered during the drilling process. For instance, the drilling structures erected during the process are made of metal, and are thus susceptible to corrosion, which in turn affects the overall drilling operation [6]. A drilling fluid additive that possesses good corrosion inhibition can efficiently address this issue. Another challenge is the excessive circulation loss of fluids into the filtrate medium, which is relatively expensive. To address this problem, a drilling fluid additive should promote good control of the circulation, and of mud cake formation and thickness [7]. Wellbore collapse also may occur due to the interaction and reaction of drilling fluids with formation fluids. The drilling fluid must contain an additive that forms a mud cake with a suitable thickness to ensure that the pipe does not become stuck and to maintain the wellbore stability [8].

Other possible scenarios in drilling operations include equipment failure during wellbore completion. Wellbore completion is the process of preparing a wellbore before the production stage, to ensure that the desired hydrocarbons flow out of the formation into the wellbore, and then out [9]. In this situation, a drilling fluid requires additives that are able to control the well and to prevent any significant damage until the equipment can be repaired [10]. Another vital aspect of a drilling fluid is its ability to control pH, rheology, and, in particular, the plastic viscosity, yield point, and gel strength. pH affects the dispersion process and can greatly affect the physical properties of the drilling mud, such as the properties of the filter cake [11].

Sharma et al. observed that tamarind gum and polyanionic cellulose showed better rheological properties and filtrate loss control performance in an oil well, and also significantly reduced formation damage [12]. Meng et al. evaluated the performance of carbon ash as an additive in comparison to a rheological modifier. Carbon ash showed a superior performance, displaying better rheological properties, satisfactory filtrate loss control, and improved wellbore stability in water-based drilling fluids [13]. Moreover, Omotioma et al. found that cashew and mango extract improved corrosion resistance of water-based drilling fluids, proving that these materials are good corrosion inhibitors, and concluded that the use of extracts of plant leaves boosted the performance of additives [14]. Similarly, Al-Hameedi et al. identified that fibrous food waste material was environmentally friendly and improved the performance of water-based drilling fluids in terms of a wide range of factors, such as better pH control, fluid loss control, mud cake thickness control, and rheological properties [15]. In addition, Al-Hameedi et al. found that biodegradable grass powder (GP) in comparison to starch, which is a commonly used additive, was batter able to control fluid circulation loss. Although starch showed better rheological properties than GP, this indicates that grass can be used in a supporting capacity in combination with starch to provide a more environmentally friendly additive [16].

Furthermore, Ismail et al. established that henna leaf extract and hibiscus leaf extract enhanced rheology and filtration properties of water-based drilling fluid in comparison to a common additive used in the industry [17]. Oseh et al. also investigated henna leaf extract in terms of its efficiency in transporting cuttings during drilling operations, and showed that henna leaf was effective, and had upgraded rheology and filtration properties under thermal aging conditions [18].

The drilling industry currently uses many additives that provide satisfactory drilling fluid performance. However, these materials have been found to be hazardous, either to the workforce operating on the site or to the environment. Substantial research has been conducted on possible alternative drilling fluid additives that fulfill two conditions: first, that the additive provides the properties required of drilling fluids; and second, that it is environmentally friendly, biodegradable, and sustainable. This paper reviews the research undertaken using various environmentally friendly waste-generated additives in water-based drilling fluids. In particular, the role of these additives on rheological properties, such as plastic viscosity, yield point, gel strength, filtrate loss, and mud cake thickness, is evaluated. Additionally, as a noteworthy aspect of drilling fluids, the impact of various nanomaterials as additives in lubricity tests us summarized.

## 2. Drilling Fluids and Rheological Properties

The principal functions of drilling fluids include removal of drill cuttings and cleaning of the wellbore, lubricating and cooling the drill bit and string, maintaining wellbore formation, and preventing well blowout [19,20]. Thus, the drilling fluid plays a significant role in the upstream oil and gas industry. As a major success factor of drilling processes, the properties of drilling fluids are constantly monitored and adjusted as recommended by the American Petroleum Institute (API) Recommended Practice 13B-1 for WBDF and Recommended Practice 13B-2 for OBDF. Based on API Recommended Practice 13B-1, the International Organization for Standardization (ISO) has prepared and outlined the ISO 101,414 under the general title of Petroleum and Natural Gas Industries—Field Testing of Drilling Fluids (API, 2009) [21]. ISO 10,414 outlines the standard procedures to regularly determine and monitor the mud properties to ensure maximum drilling performance. These procedures are improved and periodically revised with the emergence of newer studies and developments.

### 2.1. Mud Density or Weight

The mud density or weight is an important property of drilling fluids that functions to improve wellbore stability and maintain formation pressure. According to Das and Chatterjee [22], low mud density can lead to shear failure of rocks, known as a borehole breakout, which subsequently collapses the wellbore. However, Ebikapaye et al. [23] reported the possible loss of circulation, decrease in rate of penetration, and formation damage due to excessive mud density values. Thus, researchers have worked to develop a reliable route, i.e., the PSO-ANN model, to estimate the most suitable density of drilling fluids under HTHP wellbore conditions [24].

### 2.2. Plastic Viscosity

Viscosity measures the internal resistance of drilling fluids, whereas PV is the flow resistivity caused by friction between the solid particulates in drilling fluids and fluid layers [25]. PV is dependent on the viscosity of the base fluids, i.e., water and oil, and the concentration of solids. In short, an increase in mud weight or solid content in drilling fluids results in higher PV, which is undesirable because it lowers the drilling speed. The adverse effects caused by PV have been reduced by the addition of water or a thinning additive [26].

### 2.3. Yield Point

YP is defined as the measured degree of shear thinning performance of non-Newtonian drilling fluids. It is the ability to carry drill cuttings in suspension while circulating in the wellbore and out of the annulus. Hence, drilling problems such as differential sticking can be prevented [27]. According to Maiti et al., as the solid additive particles reduce in size, the YP increases [28]. This is due to the increased attractive forces between solid particles which enhance the carrying capacity of drill cuttings and cleans the wellbore.

### 2.4. Gel Strength

Gel strength (GS) measures the forces of attraction between particles in static conditions, unlike YP which measures them in dynamic conditions [29]. Therefore, gel strength refers to the ability to suspend drill cuttings during connections or other static conditions. As it increases over time, more pressure is required to overcome the accumulated gel strength and initiate the circulation [30].

### 2.5. Filtrate Loss and Mud Cake Thickness

Filtration or fluid loss measures the amount of liquid that permeates a solid mud cake formation. According to previous researchers [31], drilling fluids invade well formations in response to the greater hydrostatic pressure of fluids compared to the pore pressure. This leads to the formation of mud cakes as the pores are filled with suspended solids from the drilling mud. Consequently, the rate of filtrate loss and mud cake thickness decreases as solid concentration in drilling fluids increases. Both filtration rate and mud cake thickness are monitored properties of drilling fluids. This is because to high filtrate loss and mud cake thickness could potentially lead to sticking of the differential pipe [32]. An exceptional mud cake possesses extremely low permeability while being equally thin, tough, and compressible. Filtration control is costly due to requiring many control factors, such as concentration of drilling fluids, size and type of suspended solids, concentration of fluid loss control (FLC) additives, and thermal stability of the system [33].

## 3. Waste Derivatives in Drilling Fluids

### 3.1. Emergence of Waste Materials in the Environment

**Figure 1:**
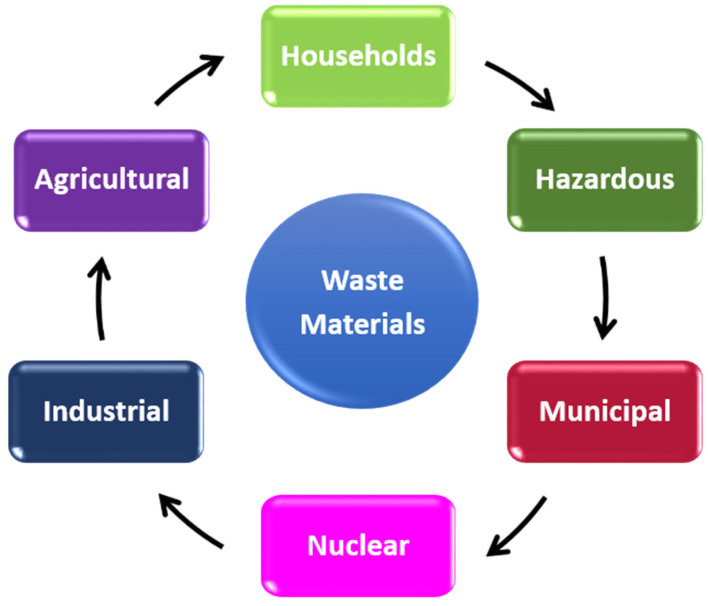
Production of various types of waste.

The global population, which is currently 7.8 billion and growing at 1.1% per year, relies on the consumption of the Earth’s natural resources [34]. Waste materials are unusable materials that have exceeded their use and been discarded. Unfortunately, an effect of this continued consumption is the proliferation in waste materials of all varieties, as shown in Figure 1 [35].

Waste material includes municipal solid waste (MSW), which comprises common items consumed and discarded by the public, and represents the fastest growing form of waste due to its prevalence in urban society [36]. In a review of solid waste management, Hoornweg and Bhada-Tata estimated that, by 2025, MSW will increase to around 1.42 kg/capita/day (2.2 billion tonnes per year), generated by 4.3 billion urban residents. The authors also estimated that 1.8 million tonnes of MSW would be generated daily in Asia [37,38].

Other types of waste are produced by a range of sources, including domestic and commercial; ash; animals; biomedical and construction industries; and sewers. These wastes may comprise industrial solid waste, biodegradable and non-biodegradable waste, and hazardous waste [39].

Some of these types of waste pose a serious threat to the environment and human health. Clinical waste, which is produced by medical clinics, hospitals, and laboratories, carries the risk of infection and may spread disease if not appropriately managed [40,41].

**Figure 2:**
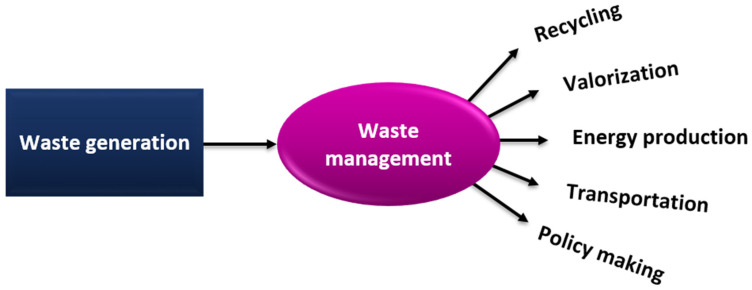
Strategies for recycling of waste materials.

Electrical and electronic waste (E-waste), from electronic equipment such as cables, wires, cords, and batteries, releases dangerous substances, and thus causes serious harm to those who contact it, particularly workers in the recycling industry [42,43]. Additionally, waste management requires recycling of hazardous waste by various approaches, as presented in Figure 2.

Food waste is a major global issue that is caused by factors including poor food processing operations and management, inadequate household planning of food consumption, and over preparation of food in the food and beverage industry [44,45]. Nevertheless, the problem of food wastage may be meaningfully addressed if the waste can be utilized and reapplied to different applications. The accumulation of wasted food in landfills results in the formation of methane gas and further pollution of the air [46,47].

### 3.2. Waste Materials in Drilling Fluids

A significant amount of research has been conducted regarding the use of food waste in the oil and gas drilling industry [48]. For instance, Al-Hameedi et al. investigated the use of mandarin peel powder (MPP) in an eco-friendly fluid additive, as an alternative to non-biodegradable additives that harm the environment. They utilized MPP as an eco-friendly alternative fluid additive in comparison to a reference polymer, PAC-LV. The MPP additive yielded better outcomes because it was able to significantly lower the pH and reduce the fluid circulation loss with a low concentration of the powder. Thus, MPP was shown to be a good additive for lowering the pH, viscosity control, and reducing circulation loss. This study encouraged the use of food waste as a suitable alternative to the non-biodegradable chemicals that are currently used in the drilling industry [49].

**Figure 3:**
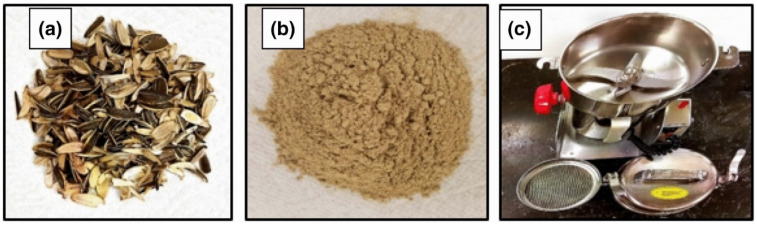
(**a**) Black sunflower shell raw waste converted into (**b**) powdered form using (**c**) a food processor [51].

Furthermore, Al-Hameedi et al. recognized that grass, hay, and palm leaves are also viable candidates. The study verified that food waste can be repurposed to promote an environmentally friendly operation of the oil and gas drilling industry [50]. Figure 3 represents the preparation of food waste as an additive for application in water-based drilling fluids.

**Figure 4:**
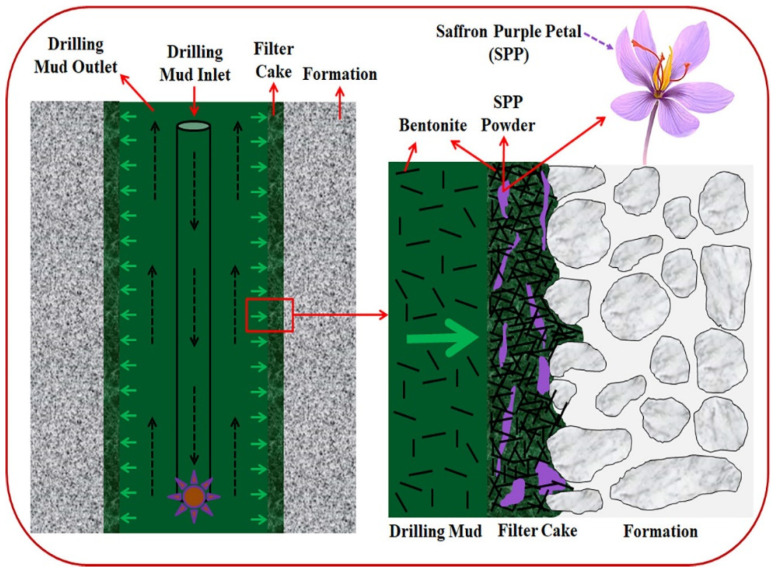
Schematic illustration of mud cake thickness and filter loss using saffron purple petals compared to bentonite based mud [56].

The oil and gas drilling industry uses additives in drilling fluids for a variety of uses, such as pH control, and to provide rheological properties, such as plastic viscosity, gel strength, and yield point [52,53]. These additives must also address issues such as circulation loss control, wellbore integrity, wellbore completion, and inhibition of corrosion to ensure a smooth drilling operation [54]. However, at present, the chemicals used for these purposes are non-biodegradable and can cause significant negative effects on the environment [55]. A sustainable solution would use chemicals that are biodegradable and do not cause any environmental damage, while simultaneously providing the desired properties of a good drilling fluid, as presented in Figure 4.

Recent studies have shown the impact of various waste-derived additives for efficient rheological properties in drilling fluids [57]. For example, Joshi et al. reported on the use of tamarind kernel powder as an alternative additive in drilling fluids. The study outlined the impact of using tamarind kernel powder on the mud density. Mud density is one of the significant properties of drilling fluids, and helps provide and regulate wellbore stability and control formation pressure. In the study, it was stated that the density of the mud sample increases with the addition of tamarind seed powder and the combination of bentonite. Increasing the concentration of tamarind seed powder resulted in a thicker mud sample and an increase in mud density. The mud density of the samples was observed to be in the range of 8.22–8.97 ppg, which has been considered to be a suitable range for use as an additive in the formulation of drilling fluids [58].

**Figure 5:**
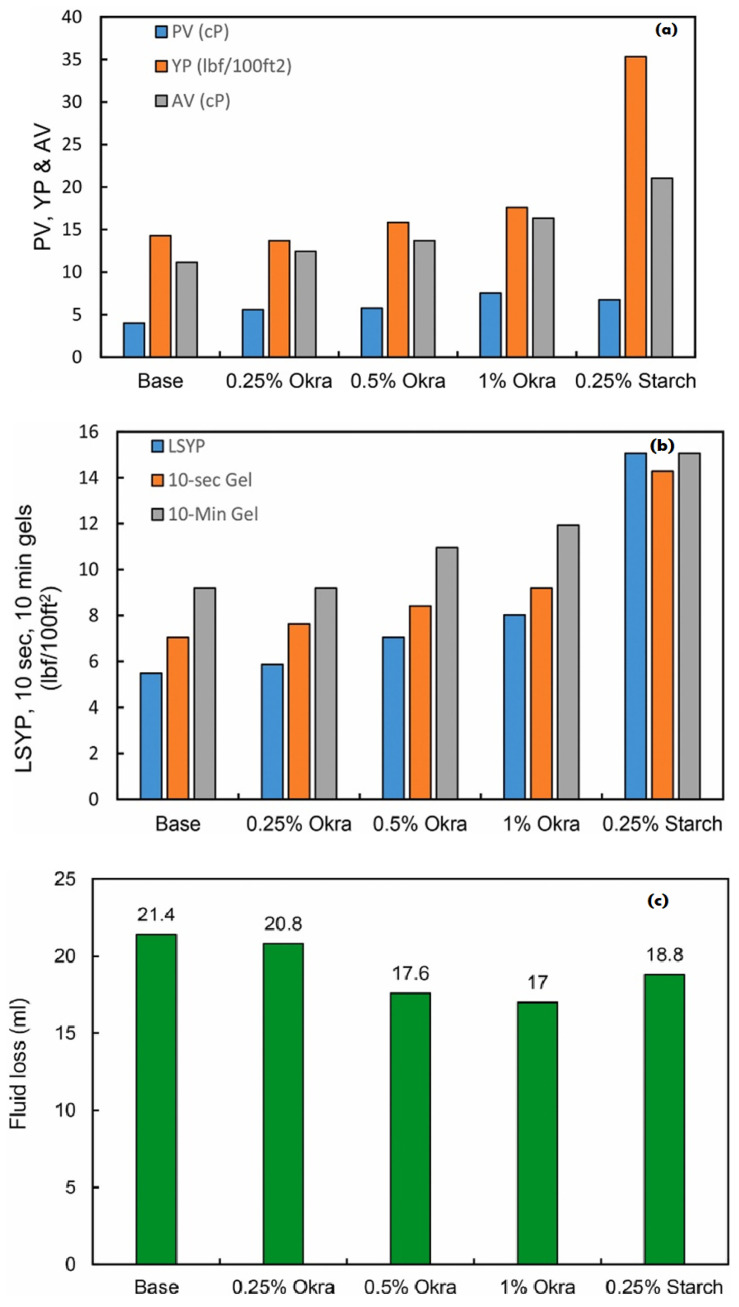

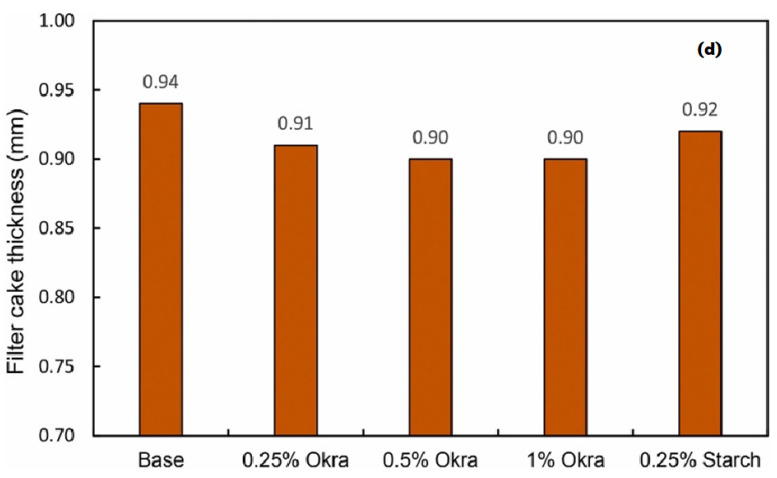
Graphical representation of okra powder as an eco-friendly additive in water-based drilling fluids. Increase in plastic viscosity, yield point, apparent viscosity, and gel strength (**a**,**b**), and decrease in fluid loss and mud cake thickness (**c**,**d**), using various additive concentrations (%) of okra powder versus starch [59].

Moreover, Murtaza et al. demonstrated the use of environmentally-friendly okra as a viable alternative additive in drilling fluids. The performance of okra as an additive was evaluated with the absence and presence of clay in drilling fluids. Comparatively, the incorporation of okra in clay-based drilling fluids presented a greater improvement in the rheological properties compared to that in clay-free drilling fluids. In clay-based drilling fluids, the addition of 2 and 3 g of okra resulted in an increase in plastic viscosity (PV) of more than 100%, compared to the addition of 2 g of starch, which only yielded a 45.7% increment. Increasing the concentration of okra also led to an increase in the yield point of drilling fluids. However, was observed that starch is more efficient in improving the yield point than okra. Fluid loss was evaluated at different concentrations and observed to be reduced at different proportions for each concentration [59]. In addition, the filter cake thickness was reduced with the addition of okra, with further reductions evident at higher concentrations, as shown in Figure 5a–d.

Similarly, Ghaderi et al. proposed sustainable saffron purple petals (SPP) as an eco-friendly alternative for additives in drilling fluids. The addition of SPP powder in drilling mud resulted in an effective increase in PV values. As the concentration of SPP powder increases, the PV value also increases. Additionally, the introduction of SPP powder to drilling mud also dramatically enhances the yield point compared to that of base mud. The incorporation of SPP powder into drilling mud demonstrated excellent filtrate loss, whereby the filtrate volume was reduced gradually with increasing concentration of SPP powder. The addition of SPP powder in the drilling mud also resulted in the reduction of mud cake thickness compared to that in base mud [56,60].

The above-mentioned studies have demonstrated the effectiveness of food waste in drilling fluid additives as a substitute for the environmentally hazardous materials currently in use within the industry [61]. In this regard, there is a need to promote the “waste to wealth” concept by studying the potential of using unused waste derivatives as additives in drilling fluids, and to address the issue by exploring the additives’ rheological properties, which ensure it is viable and cost effective [62]. Table 1 displays the role of varying waste materials used as additives for the improved rheological properties of water-based drilling fluids.

### 3.3. Bentonite in Drilling Fluids

Bentonite is a clay material that is naturally composed of sodium montmorillonite and minor quantities of minerals. It is used in a variety of applications due to its absorption and adsorption capabilities [58]. For instance, it has been used as a health remedy because it contains iron, magnesium, and calcium. In clay form, these elements are beneficial because they absorb and remove toxins from the body [80].

In the presence of water, bentonite hydrates and swells to form a thixotropic gel, i.e., a mud cake, thus protecting the well formation from invasion, which causes the loss of fluids to permeable formations. However, mud cakes formed by bentonite have been found to be highly detrimental to the productivity of drilled wells. This is due to the inefficient removal of protective mud cakes that is undertaken to restore performance of wells [81]. According to Li et al., he difficulty to remove mud cake greatly increases in deeper reservoirs. [82].

**Figure 6:**
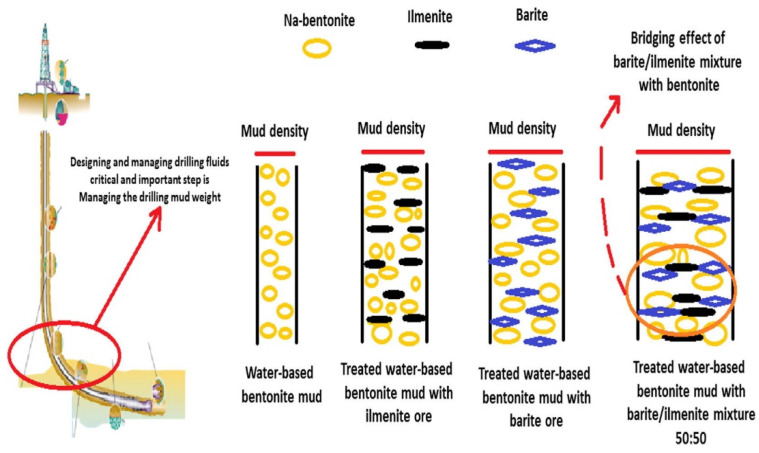
Schematic diagram of the mud weight bridging effect using bentonite, ilmenite, and barite [85].

Most importantly, because bentonite clay contains montmorillonite, a crystalline structure that forms the clay, bentonite precipitates when water is added [83]. This is beneficial in drilling fluids, in which the ability to precipitate significantly assists in reservoir formation and protection from the invasion of drilling fluids into the reservoir when exposed to water [84]. The impact of Na-bentonite as a weighing agent on mud density is compared with ilmenite and barite in Figure 6.

Magzoub et al. have studied bentonite compounds comprising different primary elements, such as calcium (Ca), potassium (K), and sodium (Na), and found that sodium bentonite is commonly utilized in drilling fluids. In contrast, calcium is rarely used due to its unsatisfactory rheological properties. However, this study used sodium to activate the calcium bentonite to improve its performance [86].

Karagüzel et al. have found that sodium and calcium bentonites in combination with soda and MgO additives show enhanced swelling properties, lower filtrate loss, and increased viscosity at favorable concentrations. An important fact to note is that although sodium and calcium bentonites were used as mud viscosifiers and fluid loss reducers, they did not qualify as good drilling fluids. This finding highlights the importance of choosing suitable additives that can effectively enhance the properties of a drilling fluid [87].

The disadvantages of bentonite have been studied, leading to the formulation of nanomaterial-based drilling fluids, and resulting in significant improvements in wellbore cleaning properties while successfully maintaining optimum viscosity and density [88]. Compared to conventional bentonite, the breakthrough study by Xie et al. have introduced the successful use of nanofluids which reduced mud filtrate and enhanced thermal conductivity as well as rheological properties of the WBDF [89].

## 4. Effects of Nanomaterials in Drilling Fluids

Nanomaterials are manufactured substances that have a size ranging from 1 to 100 nanometres (nm), and therefore are utilized in extremely small dimensions [90,91]. Nanomaterials are widely applied in various fields, such as pharmaceutical, automotive, and electronics industries and, most notably, in several areas within the chemical industry. An example of these is the drilling industry [92,93].

To facilitate the drilling of a borehole, drilling fluids imparts a vital factor for a successful drilling operation; that is, the fluids help remove the drill cuttings and fragments from the drilling area and the wellbore.

Nanomaterials have an extremely high surface area to volume ratio due to their nano-sized particles. Therefore, WBDF containing nanomaterial active agents possesses improved physical and chemical sensitivity, which enhances its performance efficiency compared to that of OBDF [94]. In addition to exhibiting advantages compared to OBDF, the improved nano-based WBDF is also cheaper and more environmentally friendly. For example, graphitic nanomaterials are excellent binding agents and have successfully been used to develop a compact, impermeable, and thinner mud cake [95]. This enables nano-pores to be physically plugged together, thereby reducing water losses during shale formations. Consequently, the use of the graphene family enhances wellbore stability [96].

**Figure 7:**
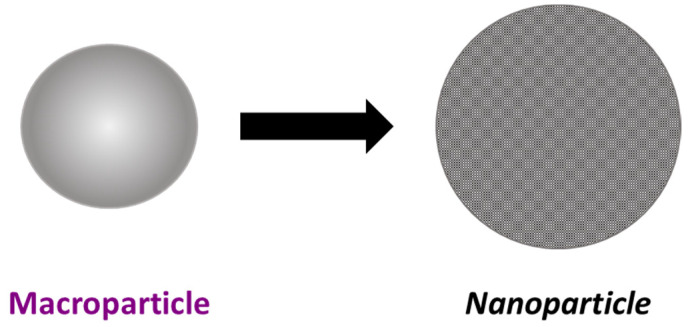
Comparison of macroparticle and nanoparticle surface area to volume ratios.

Furthermore, nanomaterials have also been used as lubricants to reduce friction between the wellbore and drill string, which consequently reduces the likelihood of a stuck pipe. Figure 7 demonstrates the increase in surface area of nanoparticles compared to macroparticles of the same volume.

The pioneering works on the significant usage of nanomaterials in drilling fluids were undertaken by Abdo et al. [97]. In enhanced oil recovery and drilling operations, Amanullah et al. reported the promising use of nanomaterials in smart fluid development due to their enhanced physio-mechanical, chemical, electrical and thermal properties [98]. At a very small concentration of nano-silica (SiO_2_), the rheological, hydraulic, and filtration properties of WBDF are effectively improved [99]. In a recent study, Karakosta et al. testified the improved drilling efficiency in the HTHP environment when metal oxide nanomaterials are used [19]. The addition of nanomaterials as additives reduced the amount of filtrate entering the reservoir, thus preventing potential damage [100]. Gautam et al. showed the positive impact of nanoparticle usage on controlling filtrate loss and mud cake thickness [101]. In summary, the use of drilling fluids improves drilling efficiency, reduces drilling costs, and is less damaging to the environment [102].

For example, Kasiralvalad et al. found that adding traces of nanomaterials to the drilling fluid, thereby making it a nanofluid, played an essential role in enhancing the mud cake quality, and reduced the sticking of the pipe to the reservoir, promoted good borehole stability and reservoir protection, and increased recovery of both oil and gas products. This was possible due to the modifications to the fluid caused by the nanoparticles, which aided in its superior performance [103].

Another reflection of this finding was provided by the study of Li et al., in which the authors established that nanomaterials helped to improve the mud cake quality and reduced loss circulation. In addition to these qualities, they also found that when nanomaterials were used as viscosifiers, emulsifiers, and lubricants, they improved the qualities of borehole cleaning, borehole stability, and reservoir protection, and enhanced oil and gas recovery [104].

**Figure 8:**
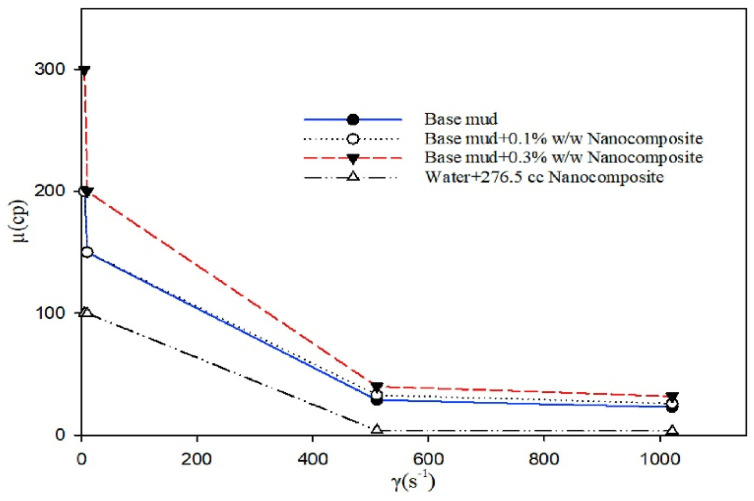
Viscosity versus shear rate using different concentrations of nanocomposite. Higher nanocomposite concentration results in a significant increase in viscosity [106].

Salih et al. emphasized the concentration of nanomaterials, and found that the rheological and filtration properties of the drilling fluid were superior at a low concentration compared to the inferior performance achieved at a higher concentration. Other researchers analyzed noteworthy parameters related to nanomaterial usage [105]. This research highlights the key factors to be considered to ensure a good performance of drilling fluids, as displayed in Figure 8.

**Figure 9:**
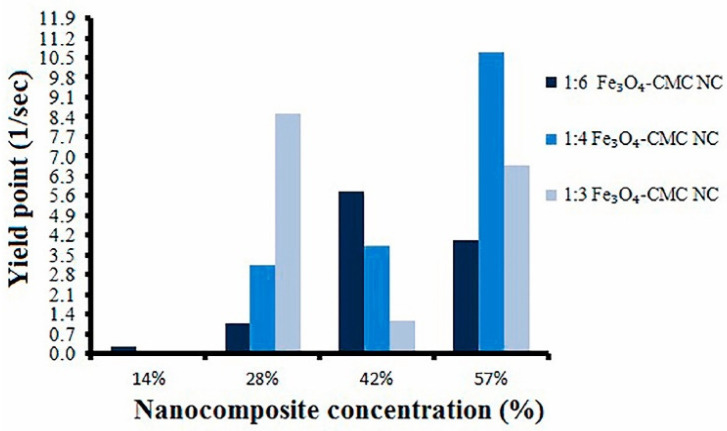
Yield point verses nanocomposite concentrations. Nanocomposite with the ratio 1:4 increased the yield point to a maximum of 50% compared to other ratios [107].

In addition to waste-derived materials, recent studies have presented nanomaterials as promising alternative for use as additives in drilling fluids. As an example, Kamali et al. assessed the effects of Fe_3_O_4_-carboxymethyl cellulose (CMC) nanocomposite as a fluid loss control additive in drilling fluids. In this study, the effect on rheological properties of drilling mud with and without salt was studied. The study observed that the utilization of the nanocomposite enhances the fluid viscosity of the drilling mud under both conditions. Based on the study results, in general, the yield point of the drilling fluid is further increased with increasing concentration of the nanocomposite. It was observed that the integration of the nanocomposite into the drilling fluid allows for the production of a thinner filter cake in comparison to the CMC mud system [107]. In addition, a reduction in filtrate loss was recorded with an increasing concentration of the nanocomposite, as seen in Figure 9.

Saboori et al. highlighted the importance of adding the appropriate concentration of additives to the drilling fluid for improved properties. They investigated the addition of varying concentrations of copper oxide (CuO), also called polyacrylamide nanocomposite, to a water-based drilling fluid. They found that increased concentration significantly minimized fluid loss and filter cake thickness in comparison to the absence of nanocomposites. They also observed higher viscosity, higher thermal conductivity, and a favorable filter cake porosity [108].

**Figure 10:**
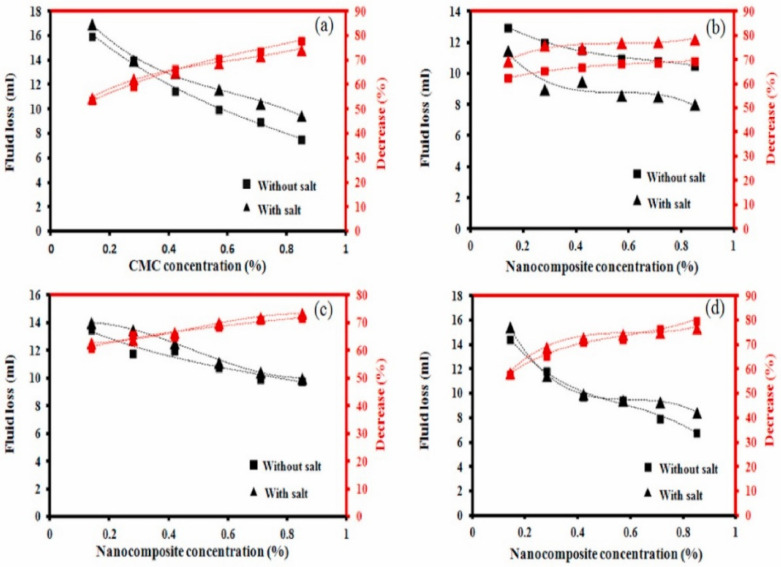
Fluid loss acquired using (**a**) CMC, (**b**) 1:6, (**c**) 1:4, and (**d**) 1:3 Fe_3_O_4_-CMC nanocomposite ratios. Higher ratios of nanocomposite show improved fluid loss volume compared to CMC [107].

Another important distinction was made by comparing drilling fluid performance between salty and non-salty water. It was found that both types displayed the best performance under certain conditions, and that a specific salt concentration may result in the best performance for both salty and non-salty water. This study again showed that additives play a vital role in the performance of a drilling fluid [109]. Figure 10 presents the effects of CMC on the reduction in filtrate loss compared to nanocomposites using various concentrations.

A compelling recent study carried out by Lekomtsev et al. utilized tools including an Extreme Learning Machine (ELM) and Particle Swarm Optimization-Least Square Support Vector (PSO-LSSVM) to investigate the effect of various nanoparticles on the filtration of a volume of drilling fluids. This research showed a decrease in the amount of filtration volume with the increase in the weight percentage of the nanoparticles. Among the parameters evaluated, the study indicated that nanoparticle concentration had the greatest impact on the filtration volume and mud cake thickness of drilling fluids [110,111].

Al-Zubaidi et al. studied nano-Iraqi clay and other nanomaterials, such as graphene and magnesium oxide (MgO), in various concentrations and separately combined with commercial nano-bentonite to observe the performance of the fluid. The addition of MgO with nanomaterials resulted in an improved filter loss and yield point. This demonstrates that a wide range of additives with appropriate concentrations can improve the performance of drilling fluids [112].

Graphenaceous materials have been extensively used in drilling fluids because they promote and enhance the rheological properties of these fluids. Kosynkin et al. found that the use of a graphene oxide additive in water-based drilling fluids improved its filtrate loss properties, significantly lowering the filter cake thickness and fluid loss, and screening enhanced shear thinning and thermal stability. It was also observed that graphene in both flake and powdered form was a contributing factor to the improved performance. This study indicates that the presence of an additive influences the properties of a drilling fluid, and shows how the form of the additive can contribute to value-added properties [113].

**Figure 11:**
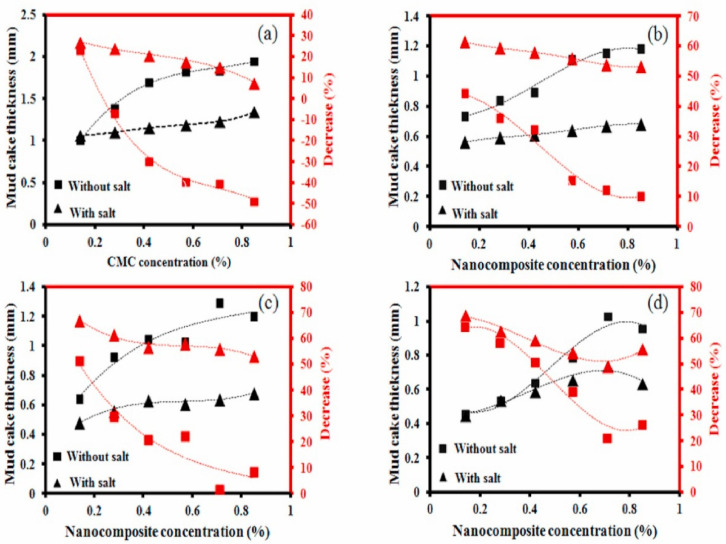
Mud cake thickness attained using (**a**) CMC, (**b**) 1:6, (**c**) 1:4, and (**d**) 1:3 Fe_3_O_4_-CMC nanocomposite ratios. The mud cake thickness was reduced by 44.27%, 51.14%, and 64.88%, respectively, as compared to CMC, which showed an increase in thickness of up to 48% [107].

Sadeghalvaad and Sabbaghi studied a TiO_2_/polyacrylamide nanocomposite as an additive in water-based drilling fluids, and found a significant reduction in fluid loss and mud cake thickness with its use, as observed in Figure 11 [114].

Patel et al. observed that conventional water drilling muds exposed to water sensitive shale cause the shale to absorb water from the mud, resulting in problems during operation. Therefore, the water-based muds must contain additives to effectively inhibit the shale. Salt compounds were used to inhibit the shale. However, the high concentrations of the salt compounds affected the surrounding ecosystems. The concentration was altered, and the shale was successfully inhibited [115]. This study demonstrates the importance of the optimal concentrations of additives in drilling fluids to achieve the superior performance of the drilling fluid without causing side effects [116].

Similarly, Qu et al. investigated polyoxyalkyleneamine (POAM) as a potential additive and found that POAM improved the shale inhibition capabilities in water-based drilling fluids [117]. As an added benefit, POAM is water soluble, has good compatibility with other additives in the drilling fluid, and is nontoxic. Subsequently, nanomaterials have been explored as potential additives to be used in drilling fluids [118].

In the drilling industry, many researchers have studied nanomaterials and found a wide range of chemicals that can improve the properties of drilling fluids. The clear advantage of using nanomaterials is that the amount required is very small [119]. Hence, the use of nanomaterials can conserve resources. The drilling industry spends millions of dollars to address situations of wellbore instability [120]. The use of nanomaterials as additives to drilling fluids should be economically sound so that resources can be conserved. For instance, nanomaterials that are used for filtration reduction, such as viscosifiers, emulsions, and clays, can decrease the rate of water penetration into shale because these nanomaterials are small enough to seal the shale, thereby strengthening the wellbore [121]. Aramendiz et al. found that SiO_2_ nanoparticles added to water-based drilling fluids enhance inhibition, and filtrate loss and rheological properties. The added benefit is that the preparation of SiO_2_ nanoparticles has a low cost due to their common methods of preparation [122].

Similarly, Taraghikhah et al. found that the optimal concentration of SiO_2_ nanoparticles is below 1% *w/v* in shale inhibition, thus constituting a very small, and hence economical, concentration [123].

Gbadamosi et al. investigated SiO_2_ nanoparticles as an additive in water-based drilling fluids. They found that SiO_2_ nanoparticles increased the viscosity of the fluid, thereby allowing it to more efficiently carry drill cuttings from the wellbore. This ensures the wellbore is clean and, therefore, does not pose challenges when the drill needs to be removed or maintenance work is required [124].

Al-Yasiri et al. also investigated the use of SiO_2_ nanoparticles with xanthan gum as a base in water-based drilling fluids, and found an increased yield point, superior hole cleaning ability, reduced filtrate loss, and more efficient lubrication of the drill bit during operation compared to drilling fluids without SiO_2_ [125].

Bayat et al. studied four nanoparticles types, namely, aluminum oxide (Al_2_O_3_), titanium dioxide (TiO_2_), SiO_2_, and CuO in bentonite, and their effects on water based drilling fluids. They found that the combined additives improved overall rheological properties and gel strength at low concentrations in comparison to the base fluid without nanoparticles [52]. This shows that additives efficiently increase rheology using a small concentration and thus conserve resources in the drilling process.

Another recent study by Medhi et al. evaluated the impact of zinc oxide (ZnO) nanoparticles on the rheological properties of non-damaging drilling fluid (NDDF). In comparison to base NDDF, NDDF incorporated with ZnO nanoparticles exhibited higher shear stress and viscosity. The addition of ZnO nanoparticles to NDDF helped overcome the issue of NDDF degradation through stabilization of viscosity at higher temperatures. Temperature sweep test measurements indicated a good operational temperature range of base NDDF was between 70 and 80 °C. NDDF containing ZnO nanoparticles exhibited improvement in fluid loss control. However, it was observed that an increase in pressure resulted in a decrease in fluid loss [126]. Table 2 summarizes numerous studies on modifications of rheological properties and reduction in filtrate loss by adding nanomaterials as additives.

### Lubricity of Drilling Fluids

One of the significant characteristics of drilling fluids is lubricity. Lubricity is required in order to reduce the friction due to the continuous contact between the wellbore and drilling string in the horizontal and directional wells [139]. There are two main aspects concerning lubricity of drilling fluids, which are referred to as torque and drag. Torque refers to the frictional resistance to the rotation of the drill string, whereas drag is described as the frictional resistance to lowering and hoisting the drill string [140]. In comparison to water-based drilling fluids, it is evident that oil-based drilling fluids provide better lubricity properties [141]. Nonetheless, water-based drilling fluid is preferred compared to oil-based drilling fluids due to the use of environmental friendly fluids in the former [142]. As a result, lubricant additives are used in water-based drilling fluids with the purpose of reducing friction between the wellbore and the drill string, lowering the probability of differential pipe sticking, and increasing the drilling rate [143].

Farahbod et al. presented a study of the thermo-physical properties of a drilling fluid incorporated with nanoparticles to examine the capability of drilling fluids to transfer heat. The use of nanoparticles, including titanium dioxide nanoparticles and CNT, were suggested because nanoparticles possess a high specific surface area, which may increase the rate of heat transfer [144]. The study observed that drilling fluids integrated with CNT exhibited a higher percentage in the ratio of convective heat to conductive heat in comparison with fluids using the titanium dioxide nanoparticles. As such, CNT are not favored for the purpose of improving the coefficient or level of convective heat transfer. Furthermore, the study observed an increase in the rate of heat transfer and the convective heat transfer coefficient with a decrease in the average size of the titanium dioxide and CNT, respectively. This indicates that nanoparticle size is a significant parameter to be considered in the utilization of nanoparticles in drilling fluids [145].

**Figure 12:**
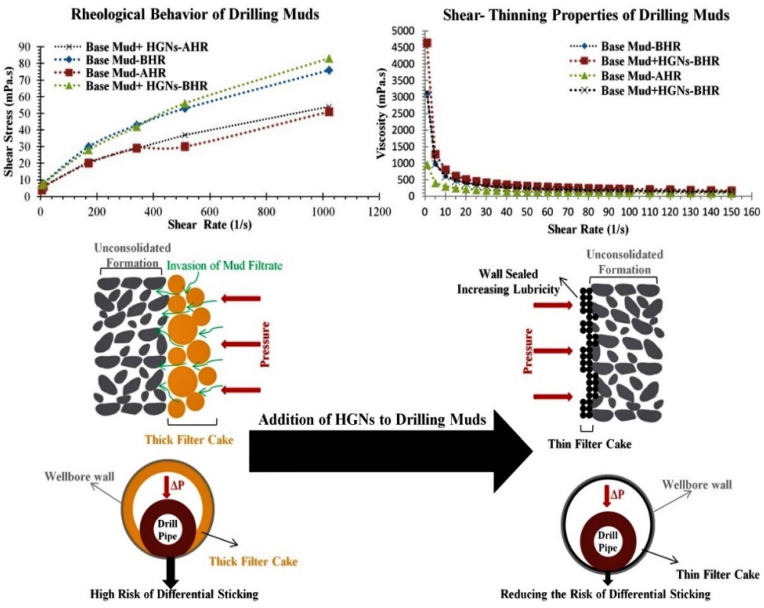
Lubricity characteristics of drilling fluids using hydrophilic gilsonite nanoparticles (HGNs) for rheological properties and assessment of the sticking of the base fluids [139].

In drilling fluids, lubricity reduces the torque and drag force [94]. Typically, lubricity of drilling fluids is measured via torque reduction, which can be determined using the coefficient of friction (CoF). The CoF is defined as the ratio of the force of the friction between two bodies and the force pressing them together [146]. Ideally, a good lubricant should possess favorable properties, including high viscosity, high lubricating film strength, low flammability, low corrosion, and high solubility, and should also be non-toxic [147,148]. The addition of a minimal quantity of lubricants is sufficient to provide drilling fluids with adequate lubricity, as shown in Figure 12.

Husin et al. reported a torque reduction of 20% with the use of a lubricant concentration of only 1% [149]. Some of the conventionally used lubricants in drilling fluids include oils, graphite, powder, surfactant, and soaps [150].

Studies have reported various types of additives used as a lubricant for drilling fluids, including modified vegetable oils and refined polyols [151]. The combination of polyols and mud changes the wetting characteristics of the mud, causing it to behave similarly to oil mud [152]. Consequently, the lubricity and shale stability of drilling mud are considerably improved. However, polyols may also change the wettability of reservoir rocks, leading to the formation of water blocks [153]. At present, polyalkylene glycols (PAGs) and polyalphaolefins (PAOs) are the most common types of lubricant used in drilling fluids. PAOs are favored in synthetic mud due to their remarkable lubricating properties, and are applied to wellbore cleaning, shale stabilization, and bit cooling and lubrication [154]. Nonetheless, PAOs possess drawbacks, including small range of viscosity and low polarity [155].

In recent decades, there has been growing interest in the use of nanomaterials as lubricant additives. This interest has been motivated by the movement in the industry toward the use of water-based drilling fluid due to the environmental concerns associated with the use of oil-based and synthetic drilling fluids [156]. The novel properties of nanoparticles offer many potential applications, particularly to the oil and gas industry. Recently, Aftab et al. demonstrated the potential use of environmentally-friendly Tween 80/ZnO nanoparticles for use in drilling fluids. The approach significantly improved the lubricity and rheological properties of the drilling fluid due to the asymmetrical morphology of the nanoparticles, which eased the rotation of metal–metal surfaces and resulted in a reduced CoF [137].

Table 3 represents the variety of nanomaterials used as lubricant in water-based drilling fluids.

## 5. Challenges and Limitations

Based on the results of recent studies, the usage of environmentally friendly additives such as bio-wastes has significantly improved the performance and functionality of drilling fluids compared to commercial GO.

**Figure 13:**
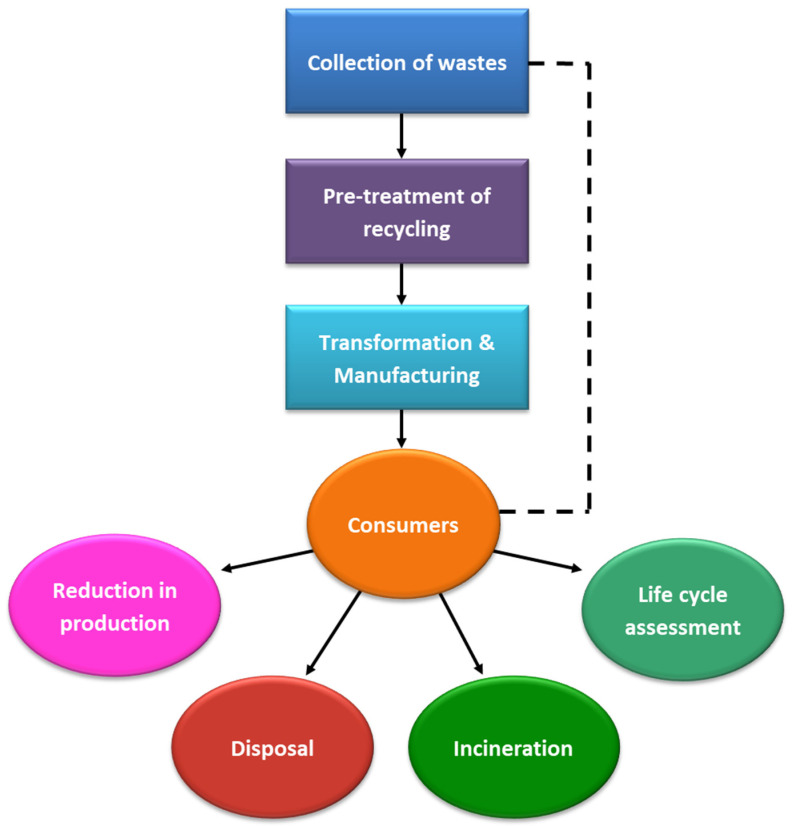
Schematic diagram of waste management approaches and their impacts (No ref).

However, several challenges must be addressed before these bio-wastes can be applied and commercialized at a larger scale in the oil and gas industry. One of the key issues is that raw waste materials and waste-derived nanomaterials may contain high impurities, thus necessitating an additional purification process. Therefore, future studies and exploration must be carried out to improve the yield and characterization of waste-derived nanomaterial production. A schematic illustration of the challenges related to the collection of waste materials and their recycling, and the interconnected role of consumers in society, is presented in Figure 13.

In addition, it is also important to examine novel waste derivatives by conducting aging tests and experimental studies under HPHT conditions to study the degradation of the environmentally friendly additive. In addition, the environmental impacts of waste-derived nanomaterials should be highlighted. These waste additives can be added to and optimized in OBDF and SBDF formulations, in addition to those of WBDF. Furthermore, a thorough comprehensive quantitative analysis of different types of green additives and their performance can be conducted to determine the best rheological improvements. Hereafter, the role of environmentally friendly waste-derived additives will have a major role in the preparation of novel green additives for drilling fluids. It is recommended that future research focuses on identifying the green additive that optimally improves the significant rheological and filtration properties of drilling fluids. Therefore, a breakthrough can be achieved by improving the efficiency of drilling operations while reducing any harmful risks to the environment and the health of personnel.

### Future Recommendations

**Figure 14:**
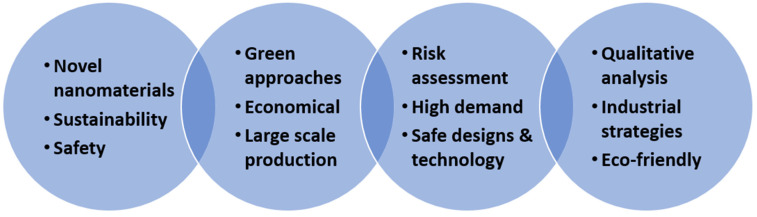
Strategies for future perspectives.

To implement the use of waste-derived materials, more research is needed to achieve a better understanding for application in the oil and gas industry. Waste materials such as food waste have the potential to be utilized as an alternative to harmful and toxic additives that are conventionally used in drilling operations. To date, a variety of waste-derived materials have been explored due to their potential use as additives. These materials include food waste, such as durian rind, and plant-derived wastes, such as black sunflower seeds. The role of waste-derived nanomaterials, and the key features with the potential to improve the efficiency of drilling performance, are presented in Figure 14.

Recommendations of this study are as follows:

- A comprehensive investigation of the interactions between waste-derived materials and content of drilling fluids, such as bentonite, should be undertaken.
- Cost-effectiveness of waste material usage requires more attention prior to commercialization to ensure consistency in generating drilling fluids with improved rheological properties.
- In-depth analysis is required to develop extensive methodologies for the production of additives based on waste-derived materials.
- Future studies should consider the analysis of the lubricity of drilling fluids using waste-derived materials. Extensive analysis should be undertaken to examine the morphological properties of drilling fluids.
- The potential to convert waste materials into nanomaterials, and the reproducibility of the conversion, should be considered for a variety of applications.
- A comprehensive quantitative analysis of nanomaterials used in drilling operations is necessary. Particular focus is required to determine optimum concentrations to improve conservation of resources.
- More studies should strive to investigate the mechanisms of interaction between nanomaterials and other additives present in drilling fluids.
- A comparison of drilling fluid optimization between water-based drilling fluids using nanomaterials, and synthetic and oil-based drilling fluids, should be undertaken. The comparison should be conducted in relation to conventional base fluids subjected to high temperature and pressure conditions.

## 6. Conclusions

The increased production of waste materials is a significant concern due to their effect on public health and the environment. Mismanagement of food waste, in particular, has become a major global issue, thus prompting the need for better solutions that use these materials in different applications. Among various applications, food waste can be considered to be a sustainable alternative for additives in drilling fluids used in the oil and gas drilling industry. Chemical additives to drilling fluids are necessary components to facilitate drilling operations by enhancing the fluids’ properties, including rheology and filtrate loss. Studies have demonstrated that waste-derived materials, including food waste, have the potential to provide an environmentally safe alternative to toxic conventional chemical additives used in water-based drilling fluids. The materials summarized in this review include food waste and waste generated from plants. The efficiency of these materials was evaluated in terms of their effects on the yield point, plastic viscosity, filtrate loss, and mud cake thickness.

Nanomaterials are viable alternative additives for drilling fluid application. Nanomaterials can be used economically due to the small concentrations required for their efficient use in drilling fluids. Based on the summarized studies, quantities less than 1 g are sufficient to generate changes in the lubricity of drilling fluids. The lubricity of drilling fluids is a property that is considered to be necessary to ensure smooth drilling operations. For water-based drilling fluids, in particular, lubricant additives are required to provide better lubrication and thus reduce friction in drilling operations.

## Figures and Tables

**Table 1:** Summary of rheological properties of waste-derived materials in drilling fluids.

Types of Wastes Materials	Process Parameters	Range of Particle Size	Amount of Waste Used (g)	Yield Point (lb/100 ft^2^)	Plastic Viscosity (cP)	Filtrate Loss (% Reduction)	References
Basil Seed Powder (BSP)	90–150 °C	5–10 µm	1	5–45 Pa	5–28 mPa.s	10.2–67.9%	[63]
Carboxymethyl cellulose carton waste (CMC)	-	-	1–5 g	-	-	0.4–10%	[64]
Wild Jujube Pit Powder (WJPP)	6.9 MPa	54, 75, 100 µm	-	1.5–2.5 Pa	3–4 mPa.s	30–47.5%	[65]
Banana Peel Powder (BPP)	-	-	6–18 g	10–16	6–12	39–54%	[66]
Black Sunflower Seeds Shell Powder	250 °F, 500 psi	52–400 µm	3.5–24.5 g	26–47	7–13	0.3–25%	[51]
Brachystegia eurycoma rice husk	-	-	20	-	-	35.62%	[67]
Detarium microcarpum rice husk	-	-	15	-	-	44.44%	[67]
Fibrous Food Waste Material (FFWM)	100 psi	2%	-	13	8	7.0 cc/30 min	[15]
Green Olive Pits’ Powder (GOPP)	-	1.5%	9	26	7	11.5 cc/30 min	[68]
Henna leaf extract	78 °F, 300 °F, 100 psi	-	10–40	33–52	23–45	29.9- 32%	[17]
Hibiscus leaf extract	78 °F, 300 °F, 100 psi	-	10–40	73–148	41–75	31.0- 35.1%	[17]
Palm Tree Leaves Powder (PTLP)	55 °C	3%	22	5	9	8.9 cc/30 min	[69]
Potato Peels Powder (PPP)	73 °F	4%	6	6	10	8.75 cc/30 min	[70]
Saffron Purple Petals (SPP)	100 psi	-	50 g	6.04–10.67 Pa	0.016–0.039 Pa.s	23–45%	[56]
Durian rind	-	44–2000 µm	5–10 ppb	2–75	10–80	17–60%	[71]
Mandarin peels powder (MPP)	-	1–4%	-	14–57	14–63	44.0–68.0%	[49]
Date Seed Powder	100 psi	300 µm	0.25–2 ppb	4	9	8–20%	[72]
Pistachio Shell Powder (PSP)	104.44 °C, 3.45 MPa	75–150 µm	5–9 g	12.2–13.5	19.8–24	15.3–44%	[73]
Soybean Peel Powder (SB)	100 psi	-	5 ppb	23	4	60%	[74]
Grass	-	35–300 µm	0.25 -1 ppb	3.5–5	8–9	11.0–14.6%	[75]
Corn Starch	170–200 °F	<125 µm	6	-	2.67–5	31%	[76]
Rice husk	-	125µm	5–20	9.56 Pa	0.008 Pa.s	16.0–42.5%	[77]
Agarwood	-	45µm, 90µm	-	22	11.9	14.0	[78]
Sawdust	70 °C	1 mm	-	-	-	8.6%	[79]
Walnut shells	-	2–6 mm	20–60	110–180	55–80	11.0–14.5%	[80]

**Table 2:** Literature studies on the effects of nanomaterials as fluid loss agents in drilling fluids.

Types of Nanomaterials	Modified Rheological Properties	Experimental Parameters	Conclusions	References
Carbon nano-tubes (CNT)	Filtration loss (API and HTHP) Shale inhibition	LPLT and HPHT 248 °F 302 °F 347 °F 392 °F	Addition of 0.8% CNT in WBDF reduced significant filtration loss in HTHP conditions.	[127]
Ferric oxide (Fe_2_O_3_)	Filtration loss (API and HTHP)	LPLT and HPHT	Addition of Fe_2_O_3_ in nanoparticles increased fluid loss at LTLP.	[128]
Graphene	Filtration loss (API) Shale inhibition	LPHT 120 °F 351 °F	Results showed 30% API filtration loss when 1–5 wt% of graphene were added to nanoparticles in 10 ppg WBDF.	[129]
MWCNT Gold nanoparticles	Filtration loss (API) Mud cake thickness	LPLT	Au nanoparticles-MWCNT at 0.005% *w/v* exhibit reduction in filtration loss by 6%.	[130]
MWCNT Graphene oxide	Filtration loss Mud cake thickness	LPLT ~100 psi	MWCNT and graphene oxide at ratio 1:1 of 0.2g each, reduces fluid loss and mud cake thickness.	[131]
Polystyrene	Filtration loss (API and HTHP) Mud cake thickness	LPLT and HPHT 24–150 °C 100–500 psi	Reduction of 50.7% and 61.1% of filtration loss for LPLT and HPHT conditions, respectively. Low permeable and thinner mud cake thickness is also observed through addition of nano-polystyrene.	[132]
Polystyrene Clay	Filtration loss (API) Yield point Gel Strength	250°F	Nanocomposite achieved a reduced API filtration loss by 22% in WBDF and showed excellent thermal stability at high temperature, 250°F.	[109]
Sepiolite	Filtration loss Mud cake thickness	HPHT 77–365 °F 100–16,000 psi	4.0 wt% of nano-sepiolite with 30–90 nm diameter showed reduced filtration loss under HPHT conditions.	[133]
GO	Filtration loss Mud cake thickness	HPHT	Graphene oxide nanosheets using >0.5 wt% improved stability by plugging and sealing of micropores. Reduction in filtration loss by up to 50% by adding 0.8 wt% of graphene oxide was observed.	[134]
Polymer-graphene oxide	Filtration loss	240 °C	Highly efficient filtration loss properties as compared to bentonite-based mud.	[135]
SiO_2_	Filtration loss Mud cake thickness	LPLT and HPHT 199 °F 1000 psi	0.7 wt% of SiO_2_ reduces filtration losses when concentration of SiO_2_ is increased. In addition, the lowest mud cake thickness (1 mm) was also obtained.	[105]
Synthetic based Acrylamide–styrene Copolymer(SBASC)	Plastic viscosity Yield point Gel strength Filtration loss	250 °F	SBASC achieved reduction in API and HTHP filtration loss by 47.5% and 38.8%, respectively.	[136]
T80ZnO	Filtration loss	API/HTHP 80–250 °F 100–500 psi	0.7g of T80ZnO mitigated API filtration loss and HTHP filtration loss by 17% and 30%, respectively.	[137]
TiO_2_- Bentonite	Filtration loss (API and HTHP) Mud cake thickness	API/HTHP	API and HTHP filtration loss reduced by 10% and 9.2%, respectively.	[138]

MWCNT = multi-walled carbon nanotubes; API = American Petroleum Institute; HTHP = high temperature high pressure; LTLP = low temperature low pressure.

**Table 3:** Role of various nanomaterials used as lubricants in drilling fluids.

Type of Nanomaterials	Range of Particle Size	Amount of Material Used (wt%)	Coefficient of Friction (CoF)	CoF Reduction (%)	References
Graphene nanoparticles	-	1–3 vol%	0.157–0.255	-	[148]
Laponite	20 nm	0–2	-	11.3–32.3	[157]
Carbon dots	1–4 nm	0.05–1.5	0.03–0.055	33	[158]
CuO nanostructures	6–60 nm	0.8	0.168–0.199	65.4–70.9	[159]
Graphene oxide	50 nm	- 0.075	0.119 19.8	- 24.3	[142] [160]
SiO_2_ nanoparticles	10–20 nm	0.013–0.53	0.24–0.38	13–25	[161]
TiO_2_/API bentonite nanocomposite (TNBT)	29 nm	0–1.0 g	0.16–0.23	33–35	[137]
Gilsonite nanoparticles	300 nm	10 g	0.15	15	[139]
Polypropylene- SiO_2_ nanocomposite	80–390 nm		0.23–0.28	20.7	[124]
SiO_2_ nanoparticles	-	0.5–1.5 ppb	0.267–0.41	3.2–12.61	[162]
Xantham gum (XC polymer), barite and lignite	10–400, 112, 63 nm	0.2–4 g	0.178–0.357	2.72–51.49	[163]
Borate nanoparticles	35–40 nm	0.01 g	0.06–0.12	69–86.5	[164]
Boron Nitride (BN) nanoparticles	250 nm	0.05–0.20 g	0.27–0.33	24–37	[165]
Iron oxide (Fe_2_O_3_) nanoparticles	-	0.05–0.20 g	0.147–0.170	43–51	[165]
MWCNT	20–40 nm	0.0095–0.38	0.15–0.30	30–50	[166]
MWCNT	-	0.01–0.04	0.07–0.15	62	[167]
SiO_2_ nanoparticles	-	0.2–0.6	0.35–0.38	-	[168]
Titanium oxide (TiO_2_) nanoparticles	-	0.2–0.6	0.31–0.34	-	[168]
Titanium oxide (TiO_2_) nanoparticles	-	0–2.625 lb/bbl	0.34–0.38	-	[169]
Titanium Nitride (TiN)	20 nm	0.0095	0.311, 0.546	46	[170]
Titanium oxide (TiO_2_) nanoparticles	-	0.5–1.0	0.36–0.40	14.3	[171]
Boron-based nanomaterial enhanced additive (PQCB)	-	1–5	-	30–80	[172]
Zinc oxide nanoparticles deposited acrylamide composite	-	0.1–1.0 g	0.21–0.28	25	[143]
Nanographene		1–5	0.07–0.16	34.6–54.6	[129]
SiO_2_ nanoparticles	1–60 nm	0.5–2.0	0.105–0.287	22.5–71.6	[123]
Palygorskite nanoparticles	10 nm–15 µm	0–8 g	0.23–0.34	68	[140]

## Data Availability

Not applicable.
